# Voltammetric and Fluorimetric Studies of Dibenzoylmethane on Glassy Carbon Electrodes and Its Interaction with Tetrakis (3,5-Dicarboxyphenoxy) Cavitand Derivative

**DOI:** 10.3390/molecules28010185

**Published:** 2022-12-26

**Authors:** László Kiss, Zoltán Nagymihály, László Kollár, Sándor Kunsági-Máté

**Affiliations:** 1 Department of Organic and Medicinal Chemistry, Faculty of Pharmacy, University of Pécs, Honvéd Street 1, H-7624 Pécs, Hungary; 2 János Szentágothai Research Center, University of Pécs, Ifjúság útja 20, H-7624 Pécs, Hungary; 3 ELKH-PTE Research Group for Selective Syntheses, Ifjúság útja 6, H-7624 Pécs, Hungary

**Keywords:** dibenzoylmethane, host–guest interaction, voltammetry, fluorescence, cavitand

## Abstract

Due to the medical importance of dibenzoylmethane, one of the aims of the study was to find an appropriate packing material and a biologically friendly co-solvent to help its introduction into living systems. Accordingly, redox properties of dibenzoylmethane were investigated on glassy carbon electrodes in acetonitrile and in 1-propanol with cyclic voltammetry, and showed a diffusion-controlled process. In the anodic window, an oxidation peak appeared at around 1.9 V in both solvents. Cycling repeatedly between 0 and 2 V, the reproducibility of this peak was acceptable, but when extending the window to higher potentials, the electrode deactivated, obviously due to electrode material. The addition of the investigated tetrakis(3,5-dicarboxyphenoxy) cavitand did not significantly change the voltammograms. Further electrochemical experiments showed that the coexistence of water in acetonitrile and 1-propanol drastically reduces the solubility of dibenzoylmethane. Moreover, very rapid electrode deactivation occurred and this fact made the use of electrochemical methods complicated. Considering that both the cavitand and dibenzoylmethane are soluble in dimethyl sulfoxide, the interaction of these species was investigated and formation of stable complexes was detected. This observation was verified with fluorescence quenching studies. The mixture of water and dimethyl sulphoxide also dramatically improved the solubility of the cavitand–dibenzoylmethane complex at high excess of water. The addition of cavitand improved the solubility of dibenzoylmethane, a property which supports the application of dibenzoylmethane in therapy.

## 1. Introduction

Dibenzoylmethane (DBM) is the diphenyl derivative of 1,3-propanedione, and this molecule as well as its derivatives have high importance in medicine due to their anticancer properties [1,2,3,4,5,6]. There are some works in the literature concerning the investigation of DBM and it proved very efficient against prostate cancer [7]. On the other hand, derivatives of DBM have favorable UV filter properties so their utilization mixed with other UV-absorbing organic molecules in sunscreen products also have practical importance [8,9,10]. Some members of the dibenzoylmethane family proved to be excellent ligands of rare earth metal ions making their sensitive fluorimetric determination possible; their usefulness can be seen in ligand sensitized processes [11]. 

DBM can be oxidized at mildly positive potentials on commonly used electrodes and reduced at mildly negative potentials on mercury electrodes [12]. This compound has an active methylene group between two carbonyl groups. Its reduction produces anion radicals and the negative charge can delocalize from one carbonyl oxygen to another, making stable the formed radical species. 

Weak interactions between molecules are very important in several aspects. Calixarenes and cavitands provide an appropriate choice for packing molecules, being scarcely soluble in water, and these molecules can be transferred in living organisms to the corresponding sites. This capability is due to the cavity synthesized by interconnecting benzene rings through methylene bridges forming a conical hydrophobic skeleton. Usually, at the upper rim, this cavity has organic functional groups on each benzene ring so their solubility and binding properties can be changed, improving the selectivity in the binding of other molecules. Due to this practical importance, this host–guest type interaction is extensively studied, where the smaller guest molecules are mainly drugs and compounds with some degree of hydrophobicity having medical effects [13,14,15,16]. 

Different types of cavitands containing four members in their cavity have been synthesized at our university. They have high binding affinity towards certain inorganic ions [17] and organic molecules [18,19,20,21]. Herein, such a four-membered cavitand modified on its upper rim with 3,5-dicarboxyphenoxy groups was investigated with respect to binding properties towards dibenzoylmethane. Usually, cavitands are excellent packing materials for packaging compounds hydrophobic in nature as they can bind with weak *π*–*π* interactions to the appropriate functional groups found in their cavities. DBM is scarcely soluble in water therefore one of the aims of this study was to find an appropriate cavitand and co-solvent to elevate its introduction into living organisms to be suitable for anticancer applications. 

## 2. Results

### 2.1. Voltammetric Characterization of Dibenzoylmethane and Effect of Some Additives

**Figure 1:**
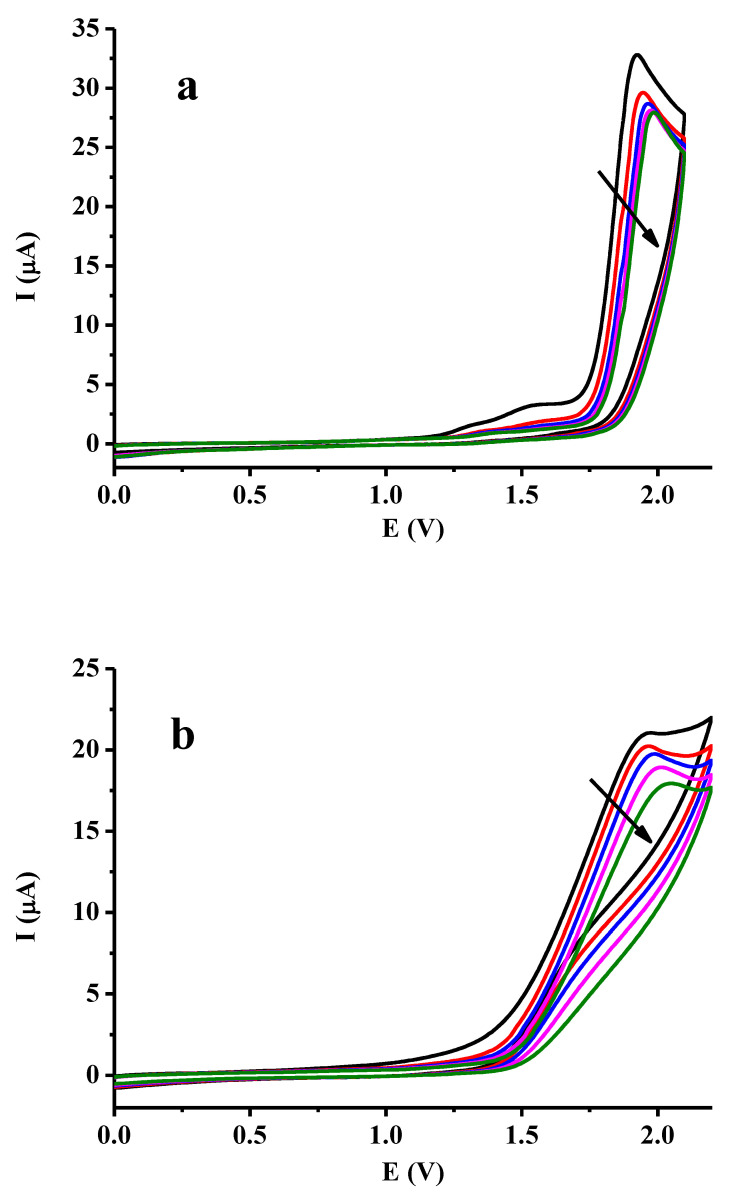
Subsequent cyclic voltammograms of 10 mmol/L dibenzoylmethane in acetonitrile (**a**), and in 1-propanol (**b**) (scan rate 0.1 V/s, supporting electrolyte 0.05 M TBAP) without polishing between scans.

In the first part of the experiments, DBM was studied in acetonitrile by cyclic voltammetry with glassy carbon electrodes, as the anodic peaks can be clearly seen on it. This was the reason for choosing glassy carbon electrodes, as with platinum electrodes worse voltammograms were acquired. Firstly, the anodic window was set between 0 and 2 V. Figure 1 shows the recorded curves, and at around 1.9 V anodic peaks showed up whose height decreased slowly by repeating the scans, which is attributable to the formation of the radical and consequent oligomer formation. When it is oxidized, a radical forms centered at one of the benzene rings, being capable of oligomerization and consequently fouling the electrode. This fact was reinforced by the voltammetric investigation of acetylacetone and benzyl. The former contains active methylene groups between two carbonyl groups without aromatic moieties. Benzyl has two benzene rings and this material is a dimer of two benzoyl groups similar to DBM. When studied with glassy carbon electrodes, only the latter gave anodic peaks a little above 2 V (not shown) indicating that the presence of aromatic rings is essential to seeing anodic peaks. The decline of anodic peaks suggests that the product of anodic oxidation adsorbs on the glassy carbon surface which is an oligomer formed from the substrate. On the other hand, the glassy carbon electrode becomes deactivated at highly positive potentials immersed in most non-aqueous solvents [22]. Part b reveals similar findings in 1-propanol, but higher background currents interfere to a large extent with the anodic peaks of DBM attributable to the solvent. The reason for studying the latter solvent was that the cavitand (see later) could be dissolved in certain concentrations of mmol/L, making it appropriate for further electrochemical investigations. In acetonitrile the cavitand was scarcely soluble. On the other hand, a protic and an aprotic solvent can be compared in this way. 

**Figure 2:**
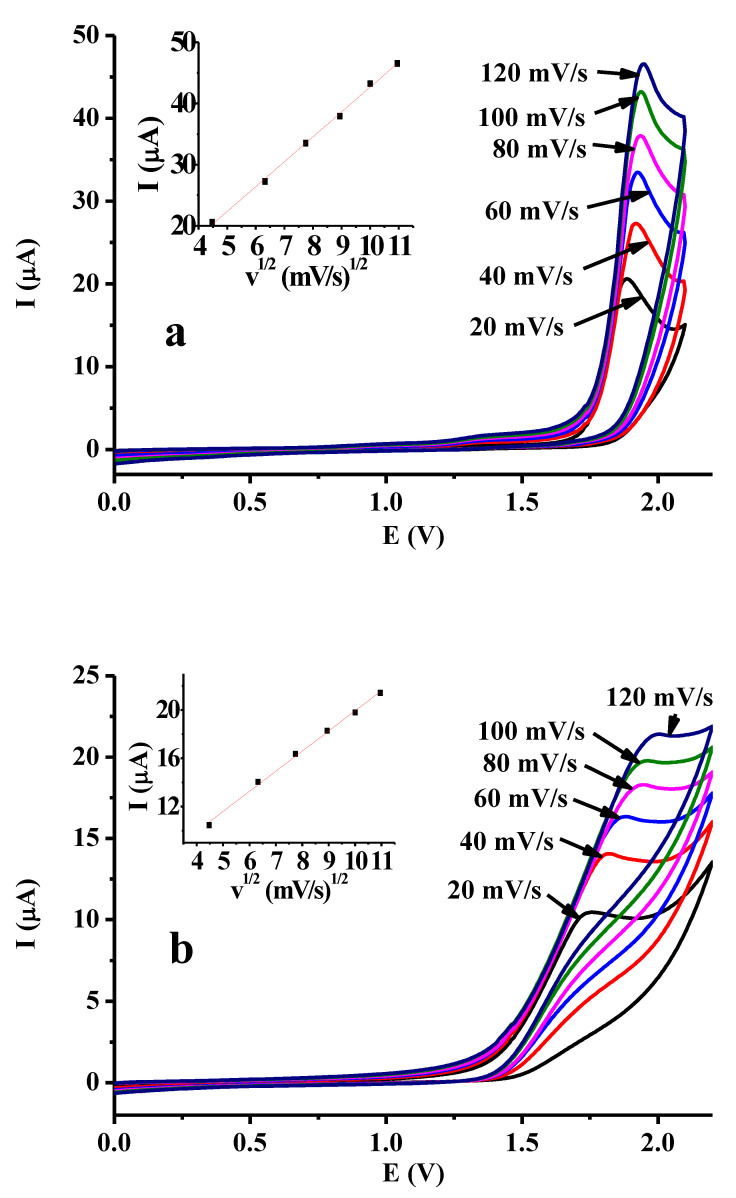
Dependence of peak currents on the square root of scan rate for 10 mM DBM in acetonitrile (**a**) and in 1-propanol (**b**) (scan rate 0.1 V/s, supporting electrolyte 0.05 M TBAP).

The dependence of peak current on scan rate provides additional information about the nature of the electrode process. As shown in Figure 1, the voltammograms were not reproducible, so the glassy carbon electrode was polished before each scan for obtaining as high a peak current as possible, so their values were very close to the diffusion-controlled signal. This must be emphasized, as from a rigorous point of view, we cannot completely measure diffusion current values as some oligomers bind by adsorption within the timescale of measuring the voltammetric signal. Figure 2 shows the obtained results when the scan rate was varied, and the *I* vs. *v*^1/2^ plots and the linear dependence indicate the negligible effect of the small disturbance of electrode deactivation occurring already by taking the scan. Basically, it proves the diffusion-controlled electrooxidation of DBM. The slope of the linear curve in the case of acetonitrile was 4.06614 and in the case of 1-propanol esd 1.66628, in accordance with solvent viscosities.

**Figure 3:**
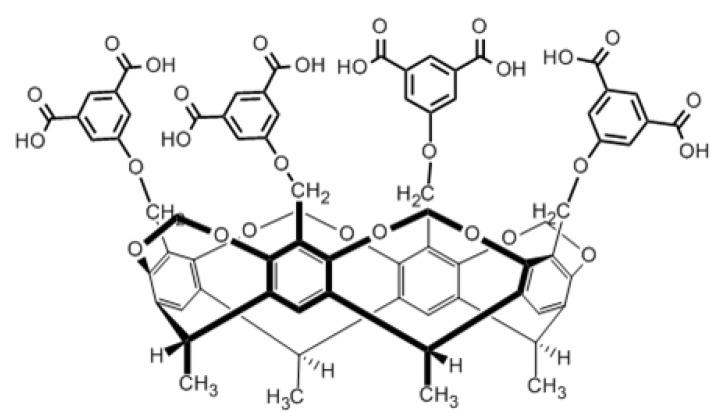
Structural formula of tetrakis(3,5-dicarboxyphenoxy) cavitand (“octacarboxy cavitand”).

**Figure 4:**
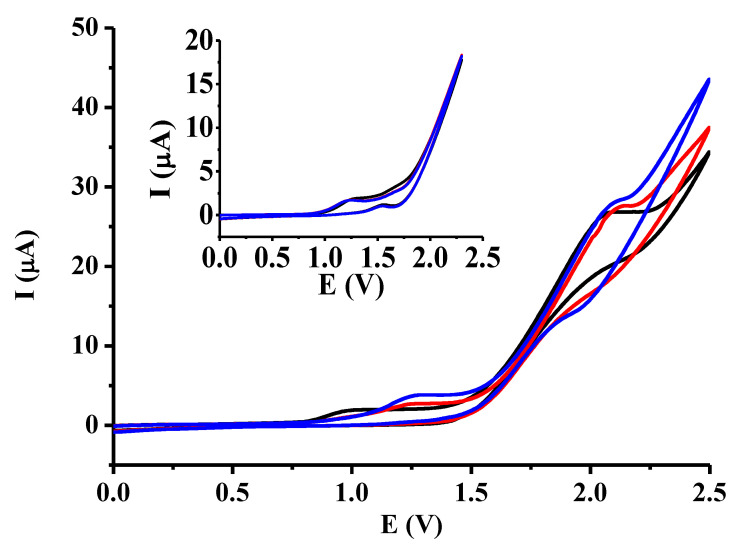
Cyclic voltammograms of 10 mM DBM in the presence of different quantities of the octacarboxyl cavitand in 1-propanol (black curve: 0 mM, red curve: 2.5 mM, blue curve: 5 mM cavitand, scan rate 0.1 V/s, supporting electrolyte 50 mM TBAP).

DBM was also investigated in presence of the octacarboxyl cavitand (Figure 3) in the selected two solvents. The effect of the addition of the cavitand in increasing portions is revealed by voltammetric curves taken in 1-propanol, and they show that the anodic peak heights of DBM increase continuously (Figure 4). The octacarboxyl cavitand has weak electroactivity between 0 and 2.5 V in 1-propanol as the inset graph of Figure 4 reveals. Repeating the cycling does not indicate electrode deactivation. However, small anodic peaks appear at around 1.6 V in the reverse sweeps as temporary electrode coverage increases. Its time duration is very small, therefore, it does not disturb the next measurement. Mainly, these small peaks appearing at around 1.6 V in the reverse scans give evidence of electroactivity of the cavitand, attributable to phenoxyl moieties. The small increases in peak currents of DBM are due to the contributions of the added cavitand.

The effect of the addition of water was also investigated in the above two non-aqueous solvents, and it was clearly seen that the solubility of DBM was drastically decreased. Recording voltammograms between 0 and 2 V in these water-containing solutions saturated with DBM showed more pronounced deactivation indicating that DBM cannot be investigated with voltammetry in environments where an aqueous phase is introduced. 

### 2.2. Titration Experiments

As dimethyl sulfoxide (DMSO) is a good solvent for many organic materials it is widely used in many applications. On the other hand, it has a very low toxicity to living organisms and is miscible completely with water, so it is used in medicine [23]. The use of DMSO in medicine dates back to 1963 as it was discovered that this liquid can penetrate the skin and biological membranes and it is capable of carrying other compounds into biological systems, especially pharmaceuticals and antioxidants. 

Neither DBM nor octacarboxy cavitand is soluble in water. DMSO readily dissolves both dibenzoylmethane and the majority of cavitands so their interaction was a focus of the studies. Firstly, the effect of the cavitand was assessed with respect to its solubilization ability towards dibenzoylmethane; thus, titration experiments were carried out. A 10 mL solution was prepared with DMSO containing 1 mM DBM and another (10 mL) containing 1 mM DBM and 1 mM cavitand. Both solutions were titrated with water till the solutions became turbid. When only DBM was present in the titration solution, 4.26 mL of water was needed until the appearance of turbidity indicating the precipitation of solid DBM from the solution phase. Surprisingly, when the cavitand was also present, turbidity could not be observed, indicating that the complex formed from the two solutes also readily dissolves in large quantity of water. Promisingly, DBM can be introduced into living organisms with the aid of DMSO and the investigated carboxy-functionalized cavitand. In other words, the favorable solvation of the complex by DMSO molecules makes the introduction of DBM into biological systems highly diluted with water.

### 2.3. Fluorimetric Investigations in Dimethyl Sulfoxide

The interaction between different compounds can be characterized with the stability constant, and fluorimetric studies are usually appropriate for this purpose, as fluorimetry can detect sensitively if binding occurs between certain molecules. As cavitands usually have high intensity fluorescence the degree of its quenching provides useful information upon addition of a guest molecule. Previous electrochemical experiments showed that study of dibenzoylmethane is complicated and leads to non-useful results upon the addition of water; thus, the choice of another method was necessary. Moreover, DMSO has high background current in the potential region where the anodic peak of DBM appears. In addition, as revealed by the titration experiments, DBM will be highly diluted by adding the necessary amount of DMSO to water to dissolve DBM completely. Therefore, the current interference caused by DMSO destroys the detection of small current signals when using voltammetric methods. 

**Figure 5:**
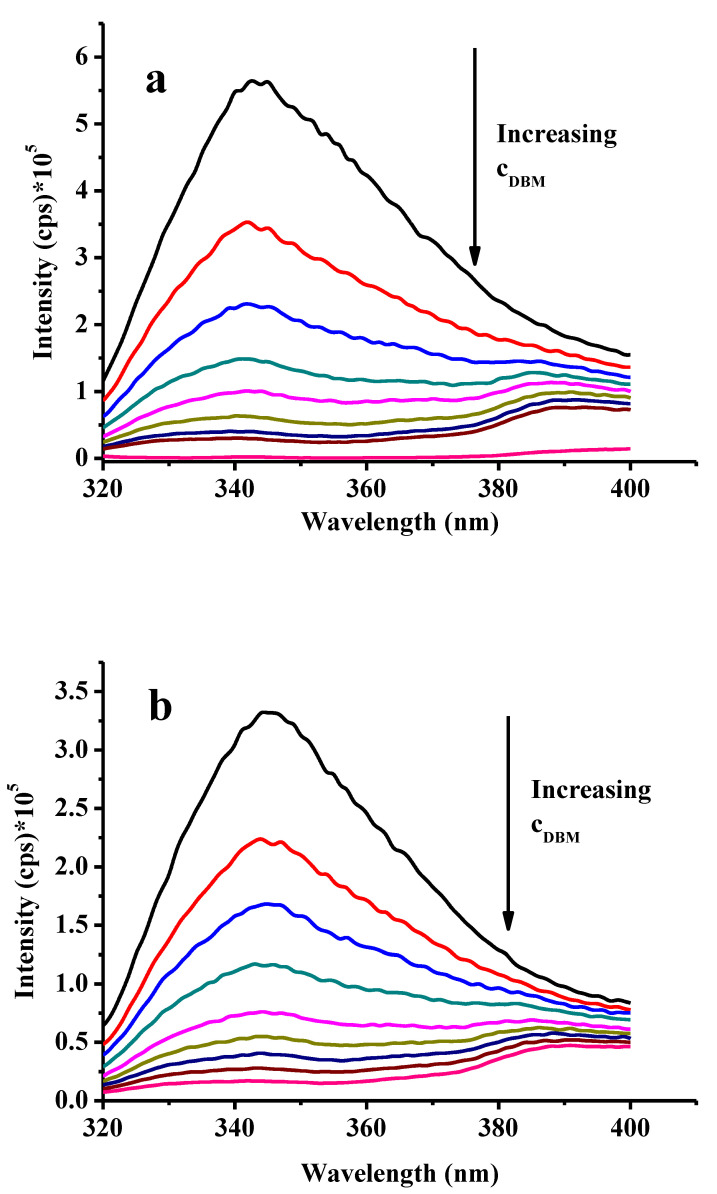
Fluorimetric curves of 10 μM calixarene upon addition of increasing amounts of DBM in DMSO (**a**) and in 50–50 *v*/*v*% DMSO–water mixtures (**b**).

In order to carry out fluorimetric examinations, data acquisitions in steady-state mode were necessary. The fluorescence method was previously used for cancer research [24]. Preliminary spectrophotometric experiments showed that the octacarboxy cavitand has a maximum absorbance at 310 nm in DMSO, therefore this wavelength was used for the excitation of cavitand. A solution series prepared with DMSO was used where concentration of cavitand was constant (10 μM) and the concentration of DBM was varied in the range of 10–80 μM. This procedure was repeated with 50–50 *v*/*v*% DMSO–water mixtures with the same concentrations, taking into account that introducing the materials into living organisms brings them in contact with an aqueous environment. The temperature for all fluorimetric measurement was set to 36 °C to adjust to the temperature of healthy mammalian organisms. The related curves are plotted in Figure 5 and the declines of fluorescence intensities upon addition of DBM suggest a strong interaction with the cavitand. Earlier, the voltammetric studies did not show any sign of interaction as the anodic peaks of DBM did not fall to lower values indicating that complexes are also electroactive.

It is clearly seen that the presence of water in the mixture diminishes the fluorescence intensities as it quenches more efficiently the fluorescence than dimethyl sulfoxide. The stability constants were also calculated using the fluorimetric curves and the Benesi–Hildebrand method. The equation used for the latter is the following:

(1) $$\frac{I_{0}}{\left(I-I_{0}\right)}=\frac{1}{A}-\frac{1}{KAc}$$

In this expression, fluorescence intensities *I*_0_ and *I* are related to the cavitand solutions in the absence and presence of the guest molecule, respectively. Furthermore, *c* is the concentration of the guest in mol/dm^3^, *A* is a constant and *K* is the stability constant (dm^3^/mol). By using this evaluation method lg*K* = 4.585 could be obtained when dimethyl sulphoxide was the solvent for the stability constant of cavitand–DBM complex and lg*K* = 4.532 when mixture of 50% dimethyl sulfoxide and 50% water was used. This indicates that the decrease in stability of the complex is not significant, thus allowing introduction into living organisms.

## 3. Materials and Methods

Dibenzoylmethane and the other chemicals were analytical grade purchased from Merck; the solvents were spectroscopic grade (VWR, Rotisolv). Throughout the electrochemical investigations glassy carbon electrodes were used as the working electrode. In the studied non-aqueous solvents, silver wire was the reference electrode, platinum wire served as counter electrode and tetrabutylammonium perchlorate (TBAP) was dissolved in solutions as the supporting electrolyte. The surface of the glassy carbon electrode was polished on a wet polishing cloth using 1 and 0.05 μm alumina powder. Finally, the polishing waste was removed from it by thorough washing with tap water and then with distilled water. After drying the electrodes with dry acetone they were embedded in the non-aqueous solutions. The three electrode system built for voltammetric measurements was connected to a potentiostat (Dropsens, Spain). 

The fluorimetric investigations were carried out with a Fluorologτ3 fluorimeter (Horiba Jobin-Yvon/SPEX; Lille, France) build with double grating excitation and single grating emission monochromators. Photoluminescence signals were detected by photomultiplier using a 1 × 1 cm quartz cuvette and right angle optical rearrangement. Excitation and emission bandwidth were set to 5 nm. For recording the absorption spectra a Specord Analytik Jena photometer was used. 

The tetrakis(3,5-dicarboxyphenoxy)cavitand was synthesized as described earlier [21].

## 4. Conclusions

The studies showed that dibenzoylmethane electrooxidation produces some deposits which readily adsorb to a glassy carbon electrode. Moreover, the hydrophobic compound dibenzoylmethane can be easily capsuled into an octacarboxy cavitand which also reacts readily on a glassy carbon electrode. Introduction in the form of a complex into living organisms is possible with the aid of low-toxicity DMSO. Due to the octacarboxy cavitand the solubility of the complex might be significantly improved and it will be a promising method in anticancer applications. After introduction into living organisms, dibenzoylmethane can get into competition with other bioactive molecules leading to the enhanced release of dibenzoylmethane from the complex. These phenomena might be the target of future works. 

## Data Availability

The data presented in this study are available on request from the corresponding author.

## Sample Availability

Samples of the compound tetrakis(3,5-dicarboxyphenoxy) cavitand are available from the authors.
